# In Vitro Benznidazole and Nifurtimox Susceptibility Profile of Trypanosoma cruzi Strains Belonging to Discrete Typing Units TcI, TcII, and TcV

**DOI:** 10.3390/pathogens8040197

**Published:** 2019-10-19

**Authors:** Susana Revollo, Bruno Oury, Andrea Vela, Michel Tibayrenc, Denis Sereno

**Affiliations:** 1 Institute of Health Diagnostic and Research Laboratory Services (SELADIS), Faculty of Pharmaceutical and Biochemical Sciences, San Andrés’ Major University, 2008 La Paz, Bolivia; susanarevollo@hotmail.com; 2 IRD, Montpellier University, InterTryp, 34000 Montpellier, France; bruno.oury@ird.fr (B.O.); andrea.vela@udlanet.ec (A.V.); 3 Center for Research on Health in Latin America (CISeAL), School of Biological Sciences, Pontifical Catholic University of Ecuador, 17 01 21 84 Quito, Ecuador; 4 IRD, Montpellier University, MiVegec, 34000 Montpellier, France; michel.tibayrenc@ird.fr

**Keywords:** chagas disease, *Trypanosoma cruzi*, benznidazole, nifurtimox, antimicrobial susceptibility test

## Abstract

We ascertain the in vitro Benznidazole (BZN) and Nifurtimox (NFX) susceptibility pattern of epimastigotes, trypomastigotes, and amastigotes of 21 *T. cruzi* strains, from patients, reservoir, and triatomine bugs of various geographic origins. Using this panel of isolates, we compute the Epidemiological cut off value (CO_wt_). Then, the frequency of the susceptible phenotype (Wild type) towards benznidazole (BZN) and nifurtimox (NFX) within this set of strains belonging to three discrete typing units (DTUs), TcI, TcII, and TcV, was deduced. We observed that the susceptibility status of individual *T. cruzi* isolates toward BZN and NFX is related to the genetic background and underlying factors that are probably related to the individual life trait history of each strain. Analyzing drug susceptibility in this conceptual framework would offer the possibility to evidence a link between isolates expressing a low susceptibility level (not wild-type) as defined by the CO_wt_ value and none-curative treatment. It will also permit us to track drug-resistant parasites in the *T. cruzi* population.

## 1. Introduction

Chagas disease or American Trypanosomosis is caused by the protozoan parasite *Trypanosoma cruzi*. Its main transmission route is vectorial, through Reduviid insects. However, other forms of transmission including the oral route, via consumption of food contaminated by triatomine feces, blood transfusions, organ transplants, or congenital route are also present [1]. Following infection, an acute phase of the disease generally manifests. During this phase, the parasite undergoes multiplication and infects local macrophages, fibroblasts, and muscle cells and can be microscopically detected in the blood. Following the control of the acute infection by a robust adaptive immune response, the disease proceeds to an indeterminate stage, which is long-lasting and asymptomatic, and is characterized by an almost undetectable parasitemia. Non-proliferative amastigote forms (hypnomastigonts) can be seen within nonphagocytic cells [2,3,4]. About one-third of the infected patients will eventually undergo a symptomatic stage characterized by cardiac and digestive clinical forms [3]. It is a lifelong infection and a major cause of morbidity and mortality, affecting 6 to 7 million people in many areas of Latin America, in Europe, and the USA, where a number of infected individuals are diagnosed within migrant populations [5,6].

The scarcity in novel agents available for antimicrobial therapy, including Chagas disease, is worsened by the development of therapy-resistant strains of microorganisms [7,8]. Until recently, two molecules, benznidazole (BZN) and nifurtimox (NFX), were available to combat Chagas disease with paucity in consensus and harmonization of standards for treatment and also well-known toxic side effects. Currently, treatment failures are frequently reported, with non-curative outcomes varying between 6% and 50% in recent clinical trials [9,10]. The extent to which the high frequency of treatment failures would be due to acquired resistance or treatments non-compliance by patients, should be established. Nevertheless, it is unlikely that the lack of drug efficacy results from the selection of genetic resistance by the use or misuse of drugs, since neither BZN nor NFX have been widely and indiscriminately used. The high variability in the efficacy of BZN and NFX treatment has been primarily attributed to the broad genetic diversity of *T. cruzi* strains. A classification based on the genetic structure of the *T. cruzi* natural populations, proposed the existence of six separated clusters or discrete typing units (DTUs), named from TcI to TcVI, in which TcV and TcVI have a hybrid evolutionary origin, with TcII and TcIII as putative parents [11]. A seventh DTU isolated from bats, namely TcBat, has been recently identified [11,12,13,14,15]. Genetic isolation between and within the DTUs has been inferred as the result of predominant clonal evolution [12,16]. In 1988, Neal and van Bueren [17] reported no correlation between the in vivo and in vitro (epimastigotes and trypomastigotes) susceptibility to BZN of several *T. cruzi* strains. A more recent work showed that the in vitro susceptibility of intracellular amastigotes of the CL and Colombiana strains are indistinguishable [18], while such strains are well known to be susceptible and highly resistant respectively, to BZN and NFX in vivo in both experimental animals and humans [19,20]. An association between DTUs and the experimental drug-treatments efficiency with BZN or Itraconazole was evidenced in an in vivo model of mice infection [21]. In addition in 1998, Revollo et al. [22] reported that epimastigote and amastigote forms of strains belonging to TcI are significantly less sensitive than those from TcII and TcV. However, another study found no correlation between *T. cruzi* susceptibility (IC50) of DTUI and DTUs II-VI on one hand, and the genetic distances deduced from RAPD (Random Amplified Polymorphic DNA) and MLEE (Multi Locus Enzyme Electrophoresis) on the other hand [23]. All these data suggest that susceptibility of *T. cruzi* in vivo infections to clinically available and experimental drugs might not only be dependent to the susceptibility of the infecting populations, but probably also on their virulence and histotropism, as well as to the PK/PD characteristics of the compound (drug accessibility). During chemotherapeutic failure, to dissect the role played by drug resistant organisms from other factors, i.e. drug disponibility, strain virulence or histotropism, it is crucial to define the susceptibility level of *T. cruzi* populations. This is obtained by computing the epidemiological cut-off value (CO_wt_) of natural *T. cruzi* populations [24]. This methodological approach has already been used to delineate wild-type (susceptible) *Leishmania* parasites from those with lower susceptibility (not wild-type forms) [25,26]. As a first step we compute the susceptibility threshold (CO_wt_) of *T. cruzi* epimastigotes, trypomastigotes, and amastigotes against BZN and NFX from a panel of previously characterized strains [22], and investigate the frequency of the sensitive (wild type) phenotype within 3 DTUs, namely TcI, TcII, and TcV. Our analysis allowed us to determine an CO_wt_ value for BZN and NFX in each parasite stage and evidenced an unequal distribution of this phenotype within the 3 DTUs studied. 

## 2. Results

### 2.1. Genetic Diversity of T. cruzi Strains under Studies

A panel of 21 strains of *T. cruzi* from diverse geographic origins and isolated from various hosts including human, animal reservoirs, and vectors, were selected (Table 1). These strains belong to three genetic lineages or discrete typing units (DTU) (TcI, TcII and TcV) among the seven currently described [27] (Table 1).

### 2.2. Epimastigote, Trypomastigote, and Amastigote Susceptibility Towards NFX and BZN

Our investigation overall evidenced that, independently from the stage considered, BZN (IC50 of 4.02 ± 2.82, 5.73 ± 3.07, and 4.00 ± 1.90 μM for epimastigotes, trypomastigotes, and amastigotes, respectively) inhibited parasite proliferation and/or survival less efficiently than NFX (IC50 of 2.46 ± 2.25, 3.60 ± 2.67 and 2.62 ± 1.22 μM for epimastigotes, trypomastigotes, and amastigotes, respectively) (Table 2 and Table 3). Another observation was that trypomastigotes appeared to possess an inherent higher capacity to resist the trypanocide effects of both NFX and BZN (Table 2 and Table 3). Specifically, for BZN, TcI epimastigotes were about three-fold less susceptible than strains belonging to TcII and TcV (Table 2). A similar ratio was also recorded for amastigotes. Such differences of IC50 were observed for NFX (Table 3). Parasites belonging to TcI were less prone to NFX-mediated growth inhibition than parasites from the two other DTUs. Altogether, it appears that parasites belonging to TcI were, in average, less susceptible to BZN- or NFX-mediated growth inhibitory effect than parasites belonging to the two other DTUs investigated by us. A box plot representation of drug susceptibility of the TcI, TcV, and TcII *T. cruzi* epimastigotes, amastigotes and trypomastigotes towards BZN and NFX is given as supplementary data S1.

**Figure 1:**
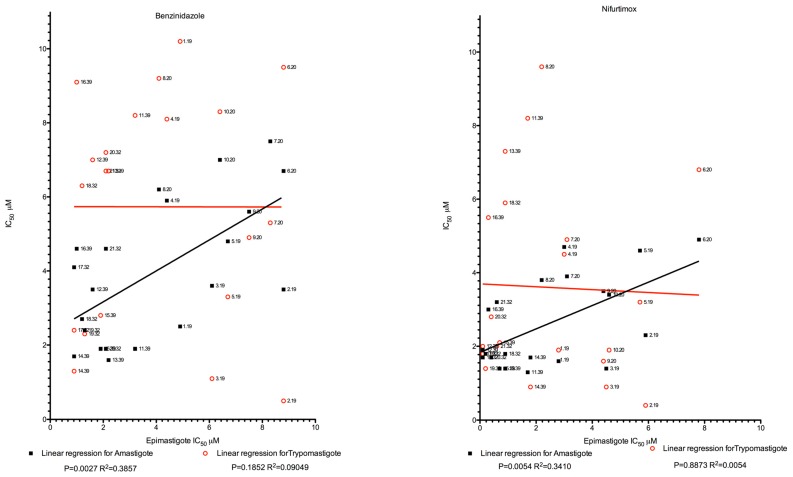
Analysis of the relationship between epimastigotes and amastigotes or trypomastigotes, for benznidazole and nifurtimox drug susceptibility. Labels of each datapoint are given.

In our experimental conditions, for both BZN and NFX, a significant correlation in drug susceptibility was observed between epimastigotes and intracellular amastigotes, but this was not found either between epimastigotes and trypomastigotes, or between amastigotes and trypomastigotes (Figure 1). This observation might be due to the fact that both epimastigote and amastigote forms are proliferative stages of the parasite, unlike the trypomastigote forms. 

### 2.3. Frequency of BZN and NFX S− (Not Wild-Type) Phenotypes

To get a clearer view of the occurrence of the susceptible phenotype in the various DTUs investigated by us, we first define its upper IC50 threshold by computing the epidemiological cut off values (CO_wt_) of BZN and NFX for the 21 strains (supplementary data S2). The ecological concept of resistance that underlies the definition of the CO_wt_, states that “a microorganism is defined as a wild type for a species by the absence of acquired and mutational mechanisms of resistance to the agent” [24]. The definition of the wild-type phenotype relies on the distribution of susceptibilities for a given compound, of unrelated strains. This makes it possible to establish the CO_wt_ value, which is the upper limit of the normal distribution of drug susceptibility for a given antimicrobial and a given species. Any strain presenting susceptibility above this value might be considered as not expressing a WT susceptibility level, we quoted them (S−), irrespective of whether the achieved level of resistance would compromise therapy.

**Figure 2:**
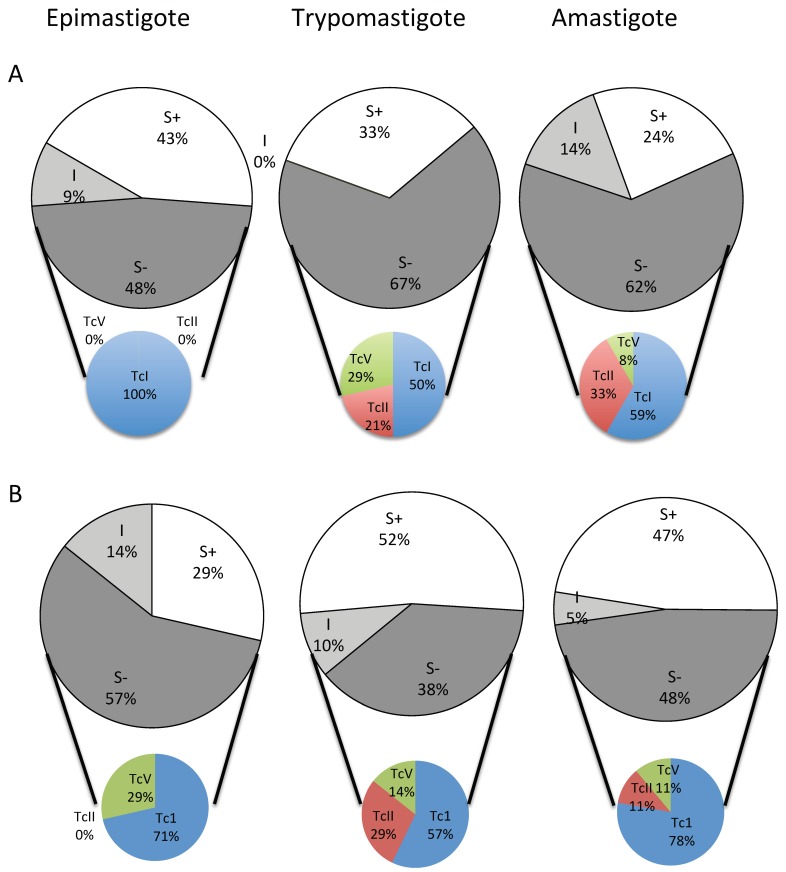
Frequency of the wild type (S+, ☐), non-wild type (S−, ■) and intermediate (I, ■) phenotype for benznidazole (**A**) and nifurtimox (**B**). Small pie chart relates the frequency of the S− phenotype in each individual DTU (Tc1 ■; TcII ■; TcV ■). Data were computed from Table 1 and Table 2.

As illustrated in Figure 2 and Table 2 and Table 3, the computed CO_w**t**_ value for the two drugs gave some relevant information. First, the CO_wt_ for BZN or NFX is higher for trypomastigotes than for epimastigotes and amastigotes. Again, this suggests that the trypomastigote stage is more tolerant to the toxic effects of both drugs. Using the computed CO_wt_ value, we then calculated the interval limit that distinguishes fully susceptible (wild-type) (S+), intermediate (I), and less susceptible (not wild-type) (S−) populations and assigned an individual phenotype to each strain (Table 2 and Table 3). A confidence interval of 85% was arbitrarily chosen to take in account the inherent variance linked to the in vitro tests used as well as the bias due to the sampling. This allowed us to gather information of the relative frequency of each phenotype within the three studied DTUs.

For both BZN and NFX (Figure 2), a none-negligible number of strains fell into the S− phenotype (between 38% to 62%). In all cases, within the S− category, parasites belonging to TcI were always more frequent than parasites belonging to the two other DTUs under study (Figure 2A,B). A striking observation is that in TcII and TcV epimastigotes, the S− phenotype for BZN is not detected, whereas it is dominant in the trypomastigote stage (21% for TcII and 29% for TcV) and the amastigote stage (33% for TcII and 8% for TcV) (Figure 2). Considering NFX, the S− phenotype comprises TcI and TcV epimastigotes, where TcI strains are dominant.

## 3. Discussion

The genetic diversity is suspected to have an impact on the susceptibility to drugs of *T. cruzi* that might impact the response to therapeutic agents. Likewise, experimental infection with *T. cruzi* has disclosed a wide variability in the cure rates with both BZN and NFX. This variability has been attributed to strain resistance against BZN and NFX [19]. Therapeutic failure encompasses a set of factors linked to the host, (among which genetic and immunologic traits), the infective agent (i.e. genetics, acquired drug resistance), the drug used (i.e., pharmacodynamics/pharmacokinetics), and the chemotherapeutic protocol. Therefore, to identify the underlying mechanisms that play a role in therapeutic failures, it is essential to univocally address the susceptibility status of the infective agent. It is therefore the in vitro antimicrobial susceptibility tests that will provide the main piece of information about the susceptibility status of the pathogen.

The main challenge for determining and comparing drug susceptibility of a large number of isolates, at various life stages of the parasite, relies on the standardization of tests. Particularly when they are performed on intracellular parasites, additional factors such as the choice of the host cells, the initial infective ratio, the methodology to ascertain parasite burden, the mode of action of the drugs tested, the parasite’s mode of invasion, and the incubation time of the parasite with the drugs can dramatically affect the outcome of the test. The epimastigote form is a parasitic stage, which develops itself only in the triatomine bugs, and does not have to face drug-mediated toxicity within infected humans under treatment. For all these reasons, epimastigotes are considered as an inadequate parasitic stage to explore the links between parasite drug resistance and therapeutic failure. However, in our experimental conditions, drug susceptibility of epimastigotes exhibits a fair correlation with drug susceptibility of intracellular amastigotes for both NFX and BZN. We noticed that epimastigotes are more susceptible to both NFX and BZN than intracellular amastigotes. This difference in drug susceptibility between parasitic stages is probably in part related to the host cell, since NFX and BZN have to cross the host cell and parasite membranes to reach amastigotes. It may also be linked to the biology and the specific physiology of the amastigote stage. The differences in the expression level and activity of nitroreductases which play a role in the bio-activation of NFX or BZN [28] and in resistance to nitroheterocyclic compounds are peculiar [29]. Trypomastigotes globally express a higher inherent capacity to resist both BZN- and NFX-mediated effects. The effect of both BZN and NFX at the Trypomastigote stage, would act only via a trypanocidal effect [30,31], but via a cumulative, trypanocidal, trypanostatic effect on replicative epimastigote and amastigote forms of the parasite.

The first clue of information on the range of susceptibility of *T. cruzi* strains is guided by the delineation of the epidemiological cut off value [24]. Such an approach has already been investigated with *Leishmania*, another trypanosomatid parasite [24]. A minimum of 20 points is required to perform such analysis, nevertheless, to get more accurate determination of the cut off, a larger number of isolates representative of the overall genetic diversity of *T. cruzi* will be beneficial. In our panel of strains and in our experimental condition, we observed an unequal distribution of the susceptible phenotype among the DTUs under study. These results suggest a direct link between the genotype of the strain and its susceptibility to drugs (BZN and NFX). Intriguingly and maybe coincidentally, the frequency of the S− phenotype recorded in the 21 isolates under study, roughly reflects the frequency of the none-curative outcome observed in some previous studies (i.e. 50%) [32,33]. Cure rates are variable but reported to be high (96%) during the acute infection and inferior to 50% in chronically infected adults [9,10]. Whatever the stage studied, strains belonging to TcI were the most frequently recorded as S−. The rate of DTU I strains with S− phenotype reaches 100% for epimastigotes. We observed that higher drug concentrations are required to kill trypomastigote forms, which do not multiply. These observations should be put in parallel with the fact that a dormant, none-replicative, form of the parasite, allows the infection to persist during treatment. While some of the amastigote parasites continue to multiply, a few of them stop their proliferation, even without drug treatment. These none-proliferating amastigotes retain their capacity to differentiate into trypomastigotes as well as their capacity to resume multiplication [34]. Interestingly, in our study, we highlight that at the trypomastigote stage, most of the strains belonging to TcI, are less susceptible (the non wild-type) to both NFX and BZN. Nevertheless, some strains of TcII and TcV which were classified as susceptible at the epimastigote stage, fall into the non wild-type category for both NFX and BZN at the trypomastigote stage. Therefore, even if susceptibility to NFX and BZN is related to the genotype, the individual life history trait of strains should represent another non-negligible factor that shapes their drug resistance potential.

Our study suggests that drug susceptibility of *T. cruzi* is related to the genotype and other additional factors linked to the life history trait of each strain. This approach will offer the possibility to perform genomic analysis on clearly defined categories, according to the drug susceptibility status of the strain. Nevertheless, our analysis does not consider the whole diversity of *T. cruzi* as it was performed on 21 isolates belonging to 3 DTU only, which still represents a limited sample. The challenge that remains is to gather data on the susceptibility of strains representative of the whole genetic, geographic and host sampling diversity of the parasite. Analysis of the life history traits of *T. cruzi* strains would provide information on the underlying factors that favored the selection of the not wild-type (S−) phenotypes within each individual DTU. These would shed light on the link between drug-resistant parasites and none-curative treatment reported during the chronic Chagas’ disease phase.

## 4. Materials and Methods

### 4.1. Parasites and Strains

The origin, host and genetic typing of the 21 *T. cruzi* strains are given in Table 1. All isolates were cloned in the laboratory by micromanipulation under microscope and the isoenzymatic genotyping was done regularly [22].

### 4.2. Parasite and Host Cell Cultivation

Epimastigote forms were grown in LIT medium supplemented with 10% heat-inactivated fetal calf serum (FCS), at 28 °C [22]. Vero cells were grown in RPMI 1640 medium, supplemented with 5% FCS, at 37 °C, in an atmosphere enriched with 5% CO2. For the production of trypomastigotes, epimastigotes were added to cells at a ratio of 10:1 in RPMI 1640 medium, supplemented with 5% heat-inactivated FCS for 24 h at 32 °C or 37 °C depending on the strain. At 24 h, cell culture was washed three times with RPMI medium to remove extracellular parasites and then incubated with RPMI medium supplemented with normal FCS serum to kill the remaining free epimastigotes until cell lysis and the release of metacyclic trypomastigotes occurred. A new cell culture was then directly infected with released trypomastigotes. 

### 4.3. Drugs

Benznidazole (BZN: N-Benzyl-2-nitro-1-imidazoleacetamide) and nifurtimox (NFX: 3-methyl-4-(5’nitrofurfurylidene-amino)-tetrahydro-4H-1-4-thiazine-1, 1-dioxide) were respectively provided by Hoffman-La Roche SA and Bayer, Argentina SA. BZN and NFX were dissolved into DMSO at a 4 mM concentration. Then, serial dilution to get final concentrations of 20 μM, 15 μM, 10 μM, 5 μM, 2 μM, 1 μM, and 0.5 μM was performed in media without fetal calf serum: LIT medium for tests on epimastigotes and RPMI medium for tests on trypomastigotes or intracellular amastigotes. IC50 was deduced according to a method that was previously described [35].

### 4.4. In Vitro Tests on Epimastigotes

The growth inhibitory effect of NFX and BZN on epimastigote forms was determined by seeding 200 μL of an epimastigotes suspension (10^6^ parasites/mL) into a 96-wells plate. After incubation for 3 or 4 days depending on DTUs, BZN or NFX was added and plates are further incubated for 72 h at 28°C. Then the inhibitory effect of BZN or NFX was measured in triplicate for each concentration by adding 1 µCi 3Hmethyl-thymidin in 20 μL, for 24 h. Cell labeling was stopped by depositing parasites on a fiberglass filter and washing them with distilled water using a Cell Harvester (Ilacon, U.K.). Radioactivity on dry filters was measured in 3 mL of scintillation fluid in a Beta LS 6000 counter (Beckman). IC50 was then determined for three independent experiments.

### 4.5. In Vitro Tests on Trypomastigotes

For trypomastigotes 200 μL of parasites suspended at a cell density of 107 parasites/mL, in RPMI 1640 medium supplemented with 5% FCS were dispatched into 96-wells plates and incubated for 24 h at 32 °C or 37 °C in the presence of 5% CO2. After addition of BZN or NFX in triplicate for each concentration, the incubation was extended of 24 h. Then 1 µCi 3H-Uracyl (20 μL) was added and radioactivity was measured 24 h later. IC50 was then determined for three independent experiments.

### 4.6. In Vitro Tests on Intracellular Amastigotes

Vero cells were seeded at a cell density of 2.5 x 105 cells/mL in culture chambers (LabTek) and cultivated in RPMI 1640 medium supplemented with 5% normal FCS for 24 h. The cellular carpet was washed to remove none-adherent cells before the addition of trypomastigotes at a parasite: host cells ratio of 10:1. After incubation for 24 h, cells were washed to remove none-internalized parasites and incubated for 72 h in the presence of NFX or BZN in triplicate for each concentration. Infected cells were then washed, fixed with methanol, and stained with Giemsa. The parasitic index PI was then calculated as follows: PI (%) = (percentage of infected cells × number of intracellular parasites/ number of infected cells in treated wells)/(percentage of infected cells × number of intracellular parasites/number of infected cells in untreated wells) × 100. IC50 value was determined for three independent experiments.

### 4.7. Cutoff Determination and Drug Sensitivity Correlation at Distinct Parasite Stages

The acronyms ECV (Epidemiological cut off value) and ECOFF (Epidemiological cut off) have been used by CLSI (Clinical and Laboratory Standard Institute) and EUCAST (European Committee on Antimicrobial Susceptibility Testing), respectively, for epidemiological cut-off values they have set from data generated in multiple laboratories. In referring to the epidemiological cut-off values established in this work, the abbreviation CO_WT_ is employed [36,37].

The epidemiological cutoff, which defines wild-type susceptible (S+) to less sensitive (not wild-type) (S−) and intermediate (I) parasite populations for benznidazole and nifurtimox, was determined with the help of a web application: cutoff finder analysis using R version 2.15.0 (2012-03-30) (http://molpath.charite.de/cutoff/). The methodology relies on the use of a mixture model of two Gaussian distributions fitted to the histogram of the drug susceptibility (IC50). The optimal cutoff was determined as the value where the probability density functions of the mixing distribution coincide [38].

The correlation analysis on NFX and BZN susceptibilities at distinct parasitic stage was performed using the linear regression function of GraphPad Prism 6.0 (GraphPad Software, La Jolla, CA, USA).

## Figures and Tables

**Table 1:** Origin, host, and genotype of the 21 cloned strains under study.

Strain Identification	Ref	DTU	Country	Host
SP104 cl1	1/19	TcI	Chile	*Mepraia spinolai*
CUTIA cl1	2/19	TcI	Brazil	*Dasiprocta aguti*
Gamba cl1	3/19	TcI	Brazil	*Didelphis azarae*
13379 cl7	4/19	TcI	Bolivia	*Homo sapiens*
OPS21 cl11	5/19	TcI	Venezuela	*Homo sapiens*
SO34 cl4	6/20	TcI	Bolivia	*Triatoma infestans*
CUICA cl1	7/20	TcI	Brazil	*Philander opossum*
P209 cl1	8/20	TcI	Bolivia	*Homo sapiens*
Esquilo cl1	9/20	TcI	Brazil	*Sciurus aestuans ingrami*
P11 cl3	10/20	TcI	Bolivia	*Homo sapiens*
SC43 cl1	11/39	TcV	Bolivia	*Triatoma infestans*
Bug2148 cl1	12/39	TcV	Brazil	*Triatoma infestans*
Bug2149 cl10	13/39	TcV	Brazil	*Triatoma infestans*
SO3 cl5	14/39	TcV	Bolivia	*Triatoma infestans*
MN cl2	15/39	TcV	Chile	*Homo sapiens*
NR cl3	16/39	TcV	Chile	*Homo sapiens*
MAS1 cl1	17/32	TcII	Brazil	*Homo sapiens*
CBB cl1	18/32	TcII	Chile	*Homo sapiens*
Tu18 cl2	19/32	TcII	Bolivia	*Triatoma infestans*
IVV cl4	20/32	TcII	Chile	*Homo sapiens*
MVB cl8	21/32	TcII	Chile	*Homo sapiens*

**Table 2:** Susceptibility to benznidazole of the 21 *T. cruzi* strains. Epi: Epimastigotes; Trypo: Trypomastigote; Ama: Amastigote stage. CO_wt_, epidemiological Cut off value. S+: Susceptible (wild-type), S−: less susceptible (not wild-type), I: Intermediate.

Ref	Epi (µM)	Status CO_wt_ = 2.68 S+<2.15>I<3.22>S−	Trypo (µM)	Status CO_wt_ = 4.01 S+<3.21>I<4.82>S−	Ama (µM)	Status CO_wt_ =2.67 S+<2.14>I<3.21>S−
1/19	4.90	S−	10.20	S−	2.5	I
2/19	8.80	S−	0.50	S+	3.5	S−
3/19	6.10	S−	1.10	S+	3.6	S−
4/19	4.40	S−	8.10	S−	5.9	S−
5/19	6.70	S−	3.30	S+	4.8	S−
6/20	8.80	S−	9.50	S−	6.7	S−
7/20	8.30	S−	5.30	S−	7.5	S−
8/20	4.10	S−	9.20	S−	6.2	S−
9/20	7.50	S−	4.90	S−	5.6	S−
10/20	6.40	S−	8.30	S−	7.0	S−
TcI mean	6.60 ± 1.75		6.04 ± 3.54		5.33 ± 1.67	
11/39	3.20	I	8.20	S−	1.9	S+
12/39	1.60	S+	7.00	S−	3.5	S−
13/39	2.20	I	6.70	S−	1.6	S+
14/39	0.90	S+	1.30	S+	1.7	S+
15/39	1.90	S+	2.80	S+	1.9	S+
16/39	1.00	S+	9.10	S−	4.6	S−
TcV mean	1.81 ± 0.85		5.85 ± 3.10		2.53 ± 1.23	
17/32	0.90	S+	2.40	S+	4.1	S−
18/32	1.20	S+	6.30	S−	2.7	I
19/32	1.30	S+	2.30	S+	2.4	I
20/32	2.10	S+	7.20	S−	1.9	S+
21/32	2.10	S+	6.70	S−	4.6	S−
TcII mean	1.52 ± 0.54		4.98 ± 2.42		3.14 ± 1.15	
Total mean	4.02 ± 2.82		5.73 ± 3.07		4.00 ± 1.90	

**Table 3:** Susceptibility to nifurtimox of the 21 strains. Epi: Epimastigote; Trypo: Trypomastigote; Ama: Amastigote stage of *T. cruzi*. **CO_wt_**, epidemiological Cut off value. S+: Susceptible (wild-type); S−: less susceptible (not wild-type), I: Intermediate.

Ref	Epi (µM)	Status CO_wt_= 1.089 S+<0.87>I<1.30 >S−	Trypo (µM)	Status CO_wt_ = 3.24 S+<2.59>I<3.88>S−	Ama (µM)	Status CO_wt_ =2.67 S+<1.70>I<2.54>S−
1/19	2.80	S−	1.90	S+	1.60	S+
2/19	5.90	S−	0.40	S+	2.30	I
3/19	4.50	S−	0.90	S+	1.40	S+
4/19	3.00	S−	4.50	S−	4.70	S−
5/19	5.70	S−	3.20	I	4.60	S−
6/20	7.80	S−	6.80	S−	4.90	S−
7/20	3.10	S−	4.90	S−	3.90	S−
8/20	2.20	S−	9.60	S−	3.80	S−
9/20	4.40	S−	1.60	S+	3.50	S−
10/20	4.60	S−	1.90	S+	3.40	S−
TcI mean	4.40 ± 1.71		3.57 ± 2.91		3.41 ± 1.25	
11/39	1.70	S−	8.20	S−	1.30	S+
12/39	0.10	S+	2.00	S+	1.90	I
13/39	0.90	I	7.30	S−	1.40	S+
14/39	1.80	S−	0.90	S+	1.70	S+
15/39	0.70	S+	2.10	S+	1.40	S+
16/39	0.30	S+	5.50	S−	3.00	S−
TcV mean	0.91 ± 0.70		4.33 ± 3.07		1.78 ± 0.63	
17/32	0.10	S+	1.80	S+	1.70	S+
18/32	0.90	I	5.90	S−	1.80	I
19/32	0.20	S+	1.40	S+	1.80	I
20/32	0.40	S+	2.80	I	1.70	S+
21/32	0.60	S+	2.00	S+	3.20	S−
TcII mean	0.44 ± 0.32		2.78 ± 1.81		2.04 ± 0.65	
Total mean	2.46 ± 2.25		3.60 ± 2.67		2.61 ± 1.22	

## Supplementary Materials

The following are available online at https://www.mdpi.com/2076-0817/8/4/197/s1. Supplementary Data S1. Box plot representation of the Benznidazole and Nifurtimox susceptibility of epimastigotes, Trypomastigotes and amastigotes as measure in the 21 strains of *T. cruzi*. Supplementary Data S2. CO_wt_ results for benznidazole (A) and nifurtimox (B) of epimastigotes, trypomastigotes and amastigotes, as computed by the online web site (http://molpath.charite.de/cutoff/).
