# Supplementary material for: In Vitro Benznidazole and Nifurtimox Susceptibility Profile of Trypanosoma cruzi Strains Belonging to Discrete Typing Units TcI, TcII, and TcV
Source: Pathogens. 2019 Oct 19;8(4):197. doi: 10.3390/pathogens8040197 (PMC6963282; doi:10.3390/pathogens8040197)
Supplement: Supplementary file 1 [file pathogens-08-00197-s001.zip › supp data/Supple data 2.pdf]

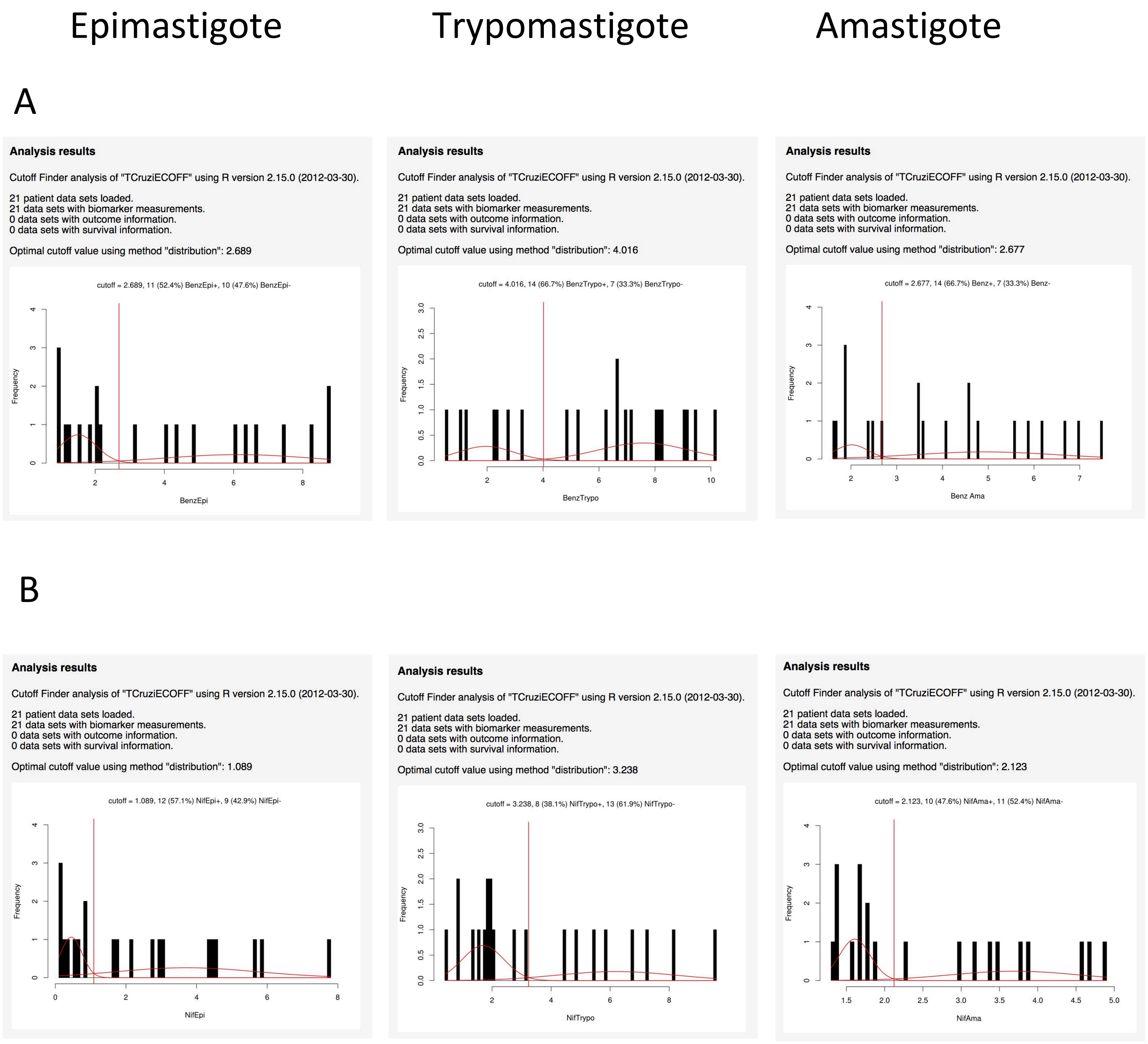

Epimastigote
Trypomastigote
Amastigote
A
Analysis results
Cutoff Finder analysis of "TCruziECOFF" using R version 2.15.0 (2012-03-30).
21 patient data sets loaded.
21 data sets with biomarker measurements.
0 data sets with outcome information.
0 data sets with survival information.
Optimal cutoff value using method "distribution": 2.689
cutoff = 2.689, 11 (52.4%) BenzEpi+, 10 (47.6%) BenzEpi-
Frequency
BenzEpi
Analysis results
Cutoff Finder analysis of "TCruziECOFF" using R version 2.15.0 (2012-03-30).
21 patient data sets loaded.
21 data sets with biomarker measurements.
0 data sets with outcome information.
0 data sets with survival information.
Optimal cutoff value using method "distribution": 4.016
cutoff = 4.016, 14 (66.7%) BenzTrypo+, 7 (33.3%) BenzTrypo-
Frequency
BenzTrypo
Analysis results
Cutoff Finder analysis of "TCruziECOFF" using R version 2.15.0 (2012-03-30).
21 patient data sets loaded.
21 data sets with biomarker measurements.
0 data sets with outcome information.
0 data sets with survival information.
Optimal cutoff value using method "distribution": 2.677
cutoff = 2.677, 14 (66.7%) Benz+, 7 (33.3%) Benz-
Frequency
Benz Ama
B
Analysis results
Cutoff Finder analysis of "TCruziECOFF" using R version 2.15.0 (2012-03-30).
21 patient data sets loaded.
21 data sets with biomarker measurements.
0 data sets with outcome information.
0 data sets with survival information.
Optimal cutoff value using method "distribution": 1.089
cutoff = 1.089, 12 (57.1%) NifEpi+, 9 (42.9%) NifEpi-
Frequency
NifEpi
Analysis results
Cutoff Finder analysis of "TCruziECOFF" using R version 2.15.0 (2012-03-30).
21 patient data sets loaded.
21 data sets with biomarker measurements.
0 data sets with outcome information.
0 data sets with survival information.
Optimal cutoff value using method "distribution": 3.238
cutoff = 3.238, 8 (38.1%) NifTrypo+, 13 (61.9%) NifTrypo-
Frequency
NifTrypo
Analysis results
Cutoff Finder analysis of "TCruziECOFF" using R version 2.15.0 (2012-03-30).
21 patient data sets loaded.
21 data sets with biomarker measurements.
0 data sets with outcome information.
0 data sets with survival information.
Optimal cutoff value using method "distribution": 2.123
cutoff = 2.123, 10 (47.6%) NifAma+, 11 (52.4%) NifAma-
Frequency
NifAma
