# Supplementary figures and images for: In Vitro Benznidazole and Nifurtimox Susceptibility Profile of Trypanosoma cruzi Strains Belonging to Discrete Typing Units TcI, TcII, and TcV
Source: Pathogens. 2019 Oct 19;8(4):197. doi: 10.3390/pathogens8040197 (PMC6963282; doi:10.3390/pathogens8040197)

# Benznidazole

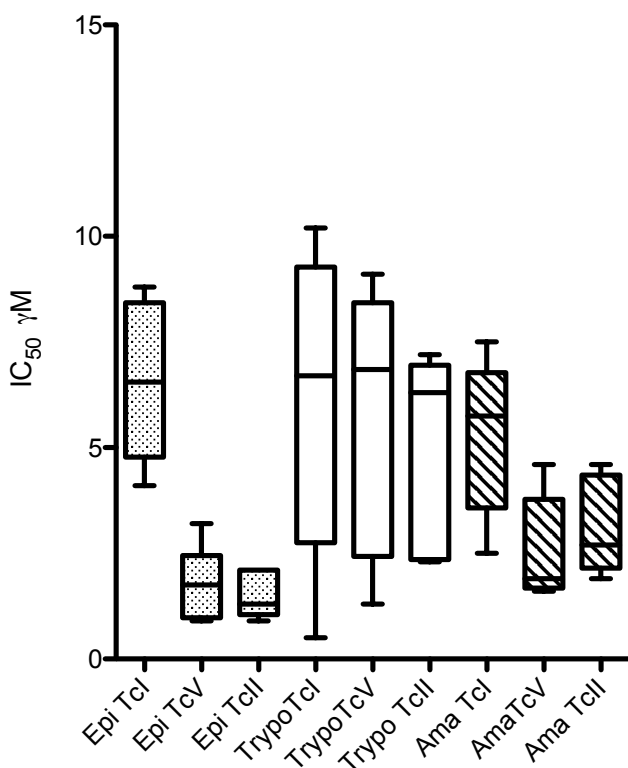

# Nifurtimox

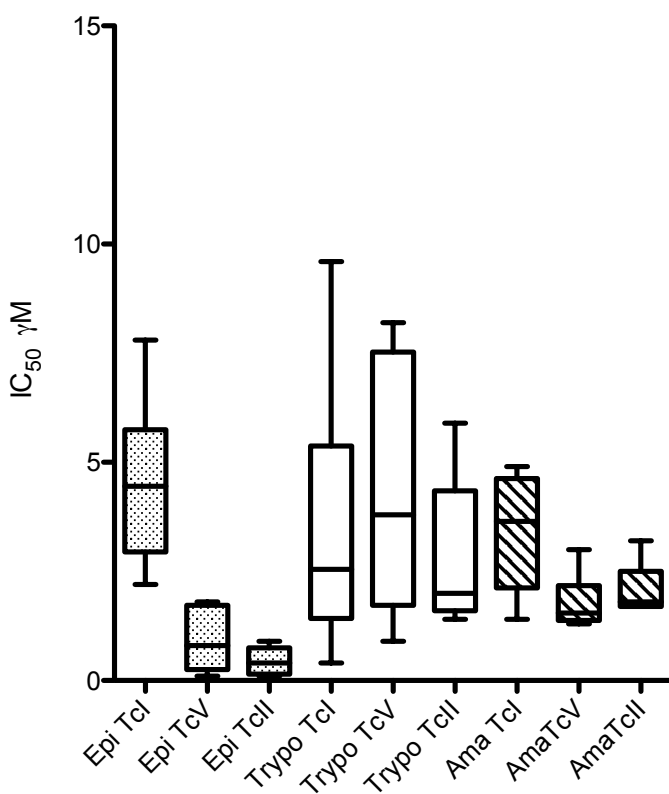

Supplement: Supplementary file 1 [file pathogens-08-00197-s001.zip › supp data/Supple data1.pdf]
